# Miscellaneous Facts and Remarks

**Published:** 1799-07

**Authors:** 


### *Miscellaneous Facts and Remarks.*

IN consequence of a hint given in our first Number, p. 83, respecting Dr. Archer's successful method of curing the croup, by means of the seneka-root, we have lately received a letter from Mr. T. Purton of Alcester.

After

After having observed that his paper on the causes and cure of that disease, lately published in the Gentleman's Magazine, is sufficiently explicit and correct, the author has favoured us with the following remarks:

"The causes which produce croup and thoracic inflammation, either partial or general, appear to me to be one and the same. The inflammation is generally of the phlegmonous kind, attacking those habits particularly, who possess an inflammatory diathesis: and, therefore, the main object, which every practioner should have in view, is, to reduce the inflammation as speedily as possible, before the exudation of the coagulable lymph takes place; for, when once the membrane is formed, I should think there will be very little chance of the patient's recovery. I had one patient only who got well, and that I thought in rather a miraculous manner. I then gave a very strong emetic solution, which brought up a quantity of ropy matter, and with excessive struggling, portions of membrane, nearly of the tenacity of the cuticle.

"Children, after they once become affected with this terrible complaint, are very liable to second attacks, whenever they catch cold, but the succeeding ones are generally less violent. I should conceive this took place upon the principle of habit, as all parts once affected with this violent disease, are certainly more open to future attacks than those which have never been affected.

"If the seneka acts as an emetic, or otherwise by its stimulus, upon the top of the trachea, so as to throw up the coagulated lymph, it may certainly sometimes cure; as nothing there but mechanical means can possibly effect a cure;—but if I may presume to give my opinion, it would be, that ninety-nine patients out of one hundred would die, or even a much greater proportion, if we imprudently waited, or were not called in, till the child was in the last stage of the disease."

---

The *Aqua Lauro-cerasi* has formerly been recommended by medical writers on the continent, as an excellent remedy (in the phrase of the humoral pathologists) for attenuating viscous blood, and resolving humours in a stagnant state, without occasioning preternatural heat, or flushings. Dr. Thilenius has lately called the attention of practitioners to this powerful remedy, which, he says, in his 'Medical and Chirurgical Remarks,' is eminently calculated to resolve thick black blood stagnating in the vessels and almost deprived of its serum. He prescribed from 40 to 50 or 60 drops of this medicine, to be taken from three to four times a day, with evident success, in cases of hypochondriasis, melancholy, mania, the blind hæmorrhoids, or suppression of the menses, obstruction of the liver, &c. provided they originated from an undue consistence of the blood. It had the effect of changing the black and heterogenous mass of the blood, so that it, after using the remedy from 14 to 24 days, acquired a more florid colour, became of a thinner consistence, and afforded the due proportion of serum. The dose of the distilled laurel-berry water was, in some instances, increased to eighty drops and upwards. Dr. Thilenius also occasionally, and with singular advantage, added a few drachms of it to the emollient clysters prescribed for his patients.

---

The *Magisterium Bismuthi*, which was first recommended by Dr. Odier, about the year 1786, appears to be either neglected or forgotten, although it is stated to be a powerful remedy in spasmodic pain of the stomach and bowels, particularly if it arise from organic debility or a relaxed and emaciated constitution. One of our correspondents affirms, that he has lately prescribed prescribed it in two or three cases of the above nature, not only with apparent, but with permanent good effect, insomuch that the cramp of the stomach, which had usually returned once or twice in the month, did not trouble his patients for six and twelve months together. He gave at first one grain of this metallic calx every half hour, for three or four times, which generally relieved the pain; but, on a second return of the cramp, he either gave a grain every quarter of an hour, with half a grain of opium, or, if the pain was not intense, a grain and a half of the bismuth every hour, without opium. In one case, four grains of the calx afforded no relief, till two more grains were given for a dose, with one grain and a half of opium, which effectually relieved the patient; so that the greatest quantity of the bismuth taken in one paroxysm, at repeated doses, did not exceed six grains.

If the magistery of bismuth could be obtained in a perfectly pure state, that is, free from all particles of lead or arsenic, which are not unfrequently combined with that semi-metal, we should not hesitate to direct even larger doses to be given, at proper intervals: but, as we are already possessed of a very excellent and powerful antispasmodic of a similar nature, the *flores zinci*, it remains to be decided which of the two deserves the preference.

---

In our second Number, p. 189, we communicated an account of Dr. Mitchill's method of applying topically, the *carbonat of potash*, for the cure of *venereal ulcerations*, with surprising expedition. We have since been informed by several of our medical friends in the metropolis, that they have observed similar good effects from the use of alkali, particularly in cases of chancres. In the course of our researches in foreign medical Journals, we found that the *caustic vegetable alkali*, (according to the "*Medical and Chirurgical Gazette*," published by Dr. Hartenkeil, of Salzburgh, No. 61, for 1792) has, even prior to that period, been employed by German practitioners, in the cure of gonorrhœa. The reviewer of Dr. Grasmeyer's method of ascertaining the difference between pus and mucus, coincides in opinion with Dr. G., that the caustic vegetable alkali changes pus into a tough matter which may be drawn in threads. He endeavours to explain the modus agendi of the alkali by the following proposition: "That, if the matter discharged in gonorrhœa, which is not essentially different from pus, be inspissated, it is then likewise deprived of its acrimony, becomes milder or less stimulant, and consequently will no longer support the inflammation: this, as the principal symptom of the disease, gradually subsiding, and the exciting cause ceasing to operate, the subsequent effect is obvious."

---

In the "Magazine for improving the Theory and Practice of Medicine and Surgery," No. I. of Vol. I. published by Dr Weikardt, of Heilbronn, who dedicates his periodical labours to the favourers as well as the opponents of the Brunonian doctrine, we met with a new mode of treating the *venereal disease*, as practised in Hindostan. The whole secret consists of a mixture of *arsenic* with the *ashes of the cedar*. We regret that we cannot state the proportion of this mixture, the doses in which it is taken, or the particular stages of lues in which it has been found effectual.

In the same Number, Dr. Weikardt has inserted an Essay "On the method of distinguishing the prevalence of the sthenic from the asthenic state in diseases;" a subject of the first importance to a Brunonian. On a future occasion, perhaps, we may lay before our readers the substance of this essay, to enable them to decide, how far Dr. W. has fulfilled the duty which he assigned to himself by engaging in this arduous task.

